# Predictors of Clinical Hematological Toxicities under Radiotherapy in Patients with Cervical Cancer—A Risk Analysis

**DOI:** 10.3390/cancers16173032

**Published:** 2024-08-30

**Authors:** Șerban Andrei Marinescu, Radu-Valeriu Toma, Oana Gabriela Trifănescu, Laurenția Nicoleta Galeș, Antonia Ruxandra Folea, Adrian Sima, Liviu Bîlteanu, Rodica Anghel

**Affiliations:** 1 Oncological Institute “Alexandru Trestioreanu” Bucharest, 252 Soseaua Fundeni, 022328 Bucharest, Romania; serban.marinescu@yahoo.com (Ș.A.M.); radu.toma@umfcd.ro (R.-V.T.); oana.trifanescu@umfcd.ro (O.G.T.); laurentia.gales@umfcd.ro (L.N.G.); antonia.folea@gmail.com (A.R.F.); 2 Faculty of General Medicine, Carol Davila University of Medicine and Pharmacy, 8 Eroilor Sanitari Street, 050474 Bucharest, Romania; 3 Department of Mathematics, Physics and Terrestrial Measurements, Faculty of Land Improvements and Environmental Engineering, University of Agronomic Sciences and Veterinary Medicine, 105 Splaiul Independentei, 050097 Bucharest, Romania; adriansima1981@gmail.com; 4 Laboratory of Molecular Nanotechnologies, National Institute for Research and Development in Microtechnologies, 126A Erou Iancu Nicolae Street, 077190 Voluntari, Romania

**Keywords:** cervical cancer, chemoradiotherapy, anemia, neutropenia, leukocytosis, thrombocytopenia

## Abstract

**Simple Summary:**

This study aims to evaluate how radiotherapy treatment for cervical cancer affects the patient’s short-term clinical status. In total, 69 cervical cancer patients were included in the study. The authors analyzed the impact of radiation on the short-term clinical adverse events and changes in blood cell counts. The results showed that factors such as age, body mass index, type of radiation therapy, and total radiation dose can predict the likelihood of a decrease in hemoglobin levels and the onset of other hematological adverse reactions. The findings of this study can help healthcare providers monitor and better care for cervical cancer patients who are at risk for these adverse reactions during treatment.

**Abstract:**

Background: Cervical cancer ranks third in frequency among female cancers globally and causes high mortality worldwide. Concurrent chemoradiotherapy improves the overall survival in cervical cancer patients by 6% but it can cause significant acute and late toxicities affecting patient quality of life. Whole pelvis radiotherapy doses of 10–20 Gy can lead to myelosuppression and to subsequent hematological toxicities since pelvic bones contain half of bone marrow tissue. Methods: A total of 69 patients with IB-IVB-staged cervical cancer have been included in this retrospective cohort study. We analyzed clinical adverse events and changes in blood cell counts (hemoglobin, neutrophils, leukocytes, and platelets) during radiation or chemoradiotherapy received at the Oncological Institute of Bucharest from 2018 to 2021. Results: Decreases in hemoglobin levels of over 2.30 g/dL during treatment were associated with BMI > 23.2 kg/m^2^ (OR = 8.68, 95%CI = [1.01, 75.01]), age over 53 years (OR = 4.60 95%CI = [1.10, 19.22]), with conformational 3D irradiation (OR = 4.78, 95%CI = [1.31, 17.40]) and with total EQD2 of over 66.1 Gy (OR = 3.67, 95%CI = [1.02, 13.14]). The hemoglobin decrease rate of 0.07 g/dL/day was related to 95% isodose volume (OR = 18.00). Neutropenia is associated frequently with gastrointestinal side effects and with the bowel and rectal V45 isodoses (OR = 16.5 and OR = 18.0, respectively). Associations of total external and internal radiation dose with the time durations calculated from the initiation of treatment to the onset of hematological adverse reactions were also obtained. The maximum drop in leukocytes was observed before day 35 from the RT initiation in patients who underwent treatment with 3D conformal radiotherapy (OR = 4.44, 95%CI = [1.25, 15.82]). Neutrophil levels under 2.2 × 10^3^/μL and thrombocyte levels under 131 × 10^3^/μL during the follow-up period were associated with a total planned dose of 54 Gy to the pelvic region volume (OR = 6.82 and OR = 6.67, respectively). Conclusions: This study shows the existence of clinical and blood predictors of hematological adverse reactions in cervical cancer patients. Thus, patients who are in a precarious clinical situation, with low hematological values (but not yet abnormal), should be monitored during days 29–35 after the initiation of RT, especially if they are obese or over 53 years of age.

## 1. Introduction

Cervical cancer ranks as the third most prevalent form of cancer among women and stands as one of the leading causes of morbidity and mortality worldwide [1]. Eurostat datasets [2], reveal that in 2020, cervical cancer ranked as the third most frequent malignancy among Romanian women, with 3380 new cases, more than 1800 deaths, and a 5-year prevalence of more than 9800 cases.

Concurrent chemoradiotherapy (CCRT) has emerged as the gold standard for locally advanced cervical cancer, having proven its role in increasing the overall survival (OS) of patients by 6% compared to radiation therapy (RT) alone [3,4]. Depending on the stage of the disease, radical hysterectomy can either precede or follow the administration of CCRT. Surgeons have the flexibility to choose an open-surgery approach, laparoscopic techniques, or robotic surgery on a case-by-case basis [5]. The acute and late toxicities of CCRT significantly impact patients’ quality of life [6] and can result in treatment delays or even discontinuation of the chosen treatment regimen [7]. The most commonly cited side effects include gastrointestinal, genitourinary, and hematological effects, primarily leukopenia and neutropenia, as well as chronic fatigue [8,9,10,11]. Myelosuppression has been observed when the pelvic bone marrow volume receives a dose of 10 to 20 Gy in patients undergoing CCRT for cervical cancer. Consequently, reducing the doses received by bone marrow tissue can potentially decrease the incidence of acute hematologic toxicity [7].

Chemotherapy (CHT) alone is known to cause hematological adverse effects, some of which can be life-threatening as is the case of febrile neutropenia [12]. On the other hand, whole pelvis RT alone can also lead to hematological toxicities (HT), due to the presence of approximately half of the body’s bone marrow tissue in the pelvic bones. The field of radiation oncology technology has made rapid advancements over the past two decades [13]. The replacement of 3D-conformal radiotherapy (3D-CRT) with intensity-modulated radiotherapy (IMRT) and volumetric arc therapy (VMAT) has significantly improved treatment outcomes and toxicity profiles for patients with locally advanced cervical cancer, establishing them as the gold standard in numerous countries [13,14].

The purpose of this paper is to explore the possibility of initiating a preventive therapy in cervical cancer patients who would present a risk of hematological adverse reactions during CCRT and in the period immediately following the administration of the treatment course by identifying some clinical or dosimetric risk factors. Thus, we investigated the association, on the one hand, between the moment of the onset of a clinical or hematological adverse reaction, the decrease in some blood count parameters, the rate of decrease in these parameters (related to time, to the dose of radiation administered and to the number of fractions administered) and, on the other hand, clinical variables (age, BMI, etc.) and planned doses to certain organs at risk.

## 2. Materials and Methods

### 2.1. Study Cohort

We performed a retrospective cohort study analyzing emerging treatment-induced clinical adverse events as well as variations in blood count (more specifically hemoglobin, neutrophils, leukocytes, and platelets), among cervical cancer patients throughout their radiation therapy or chemoradiotherapy treatment. This study included 69 patients diagnosed with cervical cancer, staged IB-IVB according to the American Joint Committee on Cancer (AJCC) 8th edition—2017 [15] and treated with neoadjuvant radiotherapy or concurrent chemoradiotherapy between 2018 and 2021.

### 2.2. Eligibility Criteria

The Ethical Review Board of the Oncological Institute of Bucharest approved the study (Authorization number 3636/22.03.2023). Following our Institute’s standard protocol, every patient participating in the study signed an informed consent form, indicating their voluntary participation in scientific research that may involve the collection of biological samples and medical data. Each patient included in the study has signed such a form.

Our study included patients aged 18 years or older, with a histologically proven diagnosis of cervical cancer We only included patients with squamous cell carcinoma and adenocarcinoma histological types, and stages IB to IVB, who received treatment at the Oncological Institute of Bucharest during the aforementioned period. Our exclusion criteria consisted of surgery as a primary treatment, metastatic disease at diagnosis, histological types other than those mentioned above, or incomplete datasets as our exclusion criteria.

### 2.3. Multimodal Treatment Strategy

The treatment protocol was determined by a multidisciplinary healthcare team in accordance with NCCN guidelines [16]. For stages IB1, IB2, and IIA1, an EBRT of the pelvis was conducted, with or without concurrent platinum-based chemotherapy, followed by intracavitary brachytherapy. For stages IB3 and IIA2, patients received CCRT (pelvic EBRT and platinum-based chemotherapy), followed by intracavitary brachytherapy. In the case of stages IIB, III, and IVA with negative lymph nodes, the chosen treatment regimen was CCRT, followed by intracavitary brachytherapy and later hysterectomy. In case of positive lymph nodes, the corresponding lymph node area was included in the irradiation field. The field was extended up to the renal vessels in case of para-aortic involvement. If involvement of the lower 1/3 of the vagina was present, the irradiation field was extended to encompass the bilateral groins. Platinum-based CHT (cisplatin or carboplatin) was administered weekly via intravenous drip followed by hydration to all patients except one. Before each CHT cycle, a comprehensive blood count and biochemical lab tests were conducted to assess renal and liver functions. CHT cycles were continued until the completion of RT if blood lab analysis tests remained normal.

### 2.4. Radiotherapy Simulation, Contouring, and Planning

Patients underwent treatment using one of two different RT modalities: 3D-CRT (two Varian linear accelerators, one commissioned for 6 MV and 15 MV energies and another one commissioned for 6 MV energy) or VMAT (one Elekta linear accelerator commissioned for 6 MV energy).

Prior to the initial CT-simulation, the patient’s rectum was emptied, and the bladder was filled. Image acquisition was performed with the patient in the supine position, with their arms positioned over their chest or head. Intravenous contrast agents were administered to enhance vascular structures and lymph node basins.

Contouring was conducted using the respective software: Eclipse™ (Varian Medical Systems Inc., Palo Alto, CA, USA) for 3D-CRT and Monaco™ (Elekta, Stockholm, Sweden) for VMAT. The contouring process involved delineating of organs at risk (OARs), such as the bladder, rectum, femoral heads, bone marrow, small bowel, and kidneys. Additionally, several target volumes were delineated: clinical target volume (CTV) 1, which consisted of the gross tumor volume (GTV), cervix uteri, and uterus with a minimum 3 cm vaginal margin from gross disease; CTV2, which included the parametria and the upper 1/3 of the vagina (or 2–3 cm caudal to the vaginal involvement); CTV3, encompassing the local lymph node areas (common, internal and external iliac, presacral, and obturator); a fourth CTV was added for para-aortic lymph node delineation if the aforementioned lymph node basin was at risk (CTV LO). For each CTV, planning target volumes (PTVs) were defined as follows: PTV1 with a 1.5 cm margin around CTV1, PTV2 with a 1 cm margin around CTV2, PTV3 with a 0.7 cm margin around CTV3, and the PTV corresponding to the para-aortic lymph node CTV, referred to as PTV LO, with a 0.7 cm margin around it.

**Figure 1:**
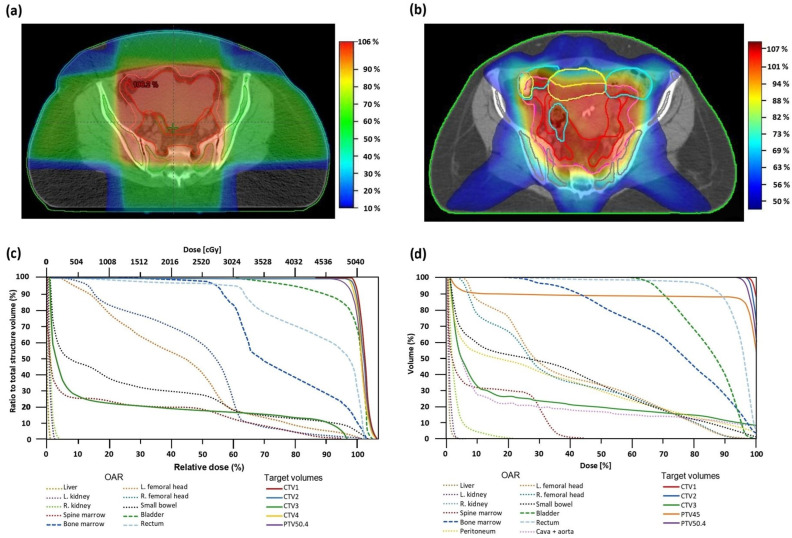
Axial planning CT sections of the pelvis with isodose levels depicted in color-wash and the corresponding dose–volume histograms (DVH) of cervical cancer patients treated with radiation therapy at the Oncological Institute of Bucharest. (**a**) The 3D-CRT treatment plan; (**b**) VMAT treatment plan; (**c**) 3D-CRT treatment plan DVH; (**d**) VMAT treatment plan DVH. Still images obtained from Eclipse™ (Varian Medical Systems Inc., Palo Alto, CA, USA) (**a**,**c**) and Monaco™ (Elekta, Stockholm, Sweden) (**b**,**d**).

Treatment setup was checked on a regular basis using cone-beam CT. OAR dose constraints were followed as they appear in QUANTEC [17]. Illustrated in Figure 1 are typical RT plans for cervical cancer patients in 3D-CRT (Figure 1a,c) and VMAT (Figure 1b,d).

The regimen and timing of initiation of the high-dose rate (HDR) brachytherapy were tailored, in particular depending on the response to external beam radiotherapy and the patient’s overall condition. Computed tomography was used to develop the brachytherapy treatment plan. Typically, and only with few exceptions, 2–5 fractions of 7.5 Gy were administered at 1–2 weeks over several weeks, the distances between administration sessions being determined by the patients’ recovery after invasive maneuvers.

### 2.5. Statistical Analysis

#### 2.5.1. Primary Variables

In this investigation, we employed a combination of primary and secondary variables. The values of the primary variables were extracted from medical records, while the secondary variables were established based on the primary variables, as demonstrated below. We utilized 5 categories of data (see Table 1) and denote the index of these categories as l. Within each category, we denote the quantitative variables by $x_{i}^{\left(l\right)}$ and the qualitative variables as follows: the binary ordinal variables are denoted by $y_{j}^{\left(l\right)}$, and the multinomial variables are denoted by $z_{k}^{\left(l\right)}$.

A comprehensive list of all the variables corresponding to each category l can be found in Tables S1–S4 of the Supplementary Materials.

In this paper, we will use the terms “time period” or “time interval” to describe the duration between the occurrence of an adverse event and radiotherapy completion. An event is an adverse reaction of the following types: general (such as fatigue or fever), digestive (for example diarrhea), genitourinary (for example dysuria) or hematological quantified by the decrease in a blood count variable (sometimes below the lower limit of the range of normal values).

#### 2.5.2. Variables Statistics

We calculated various descriptive statistics for all primary variables, including the mean value (with standard error), median, variation, standard deviation, extremes, amplitude, and distribution deviation. Additionally, we computed the percentile values of 25%, 33%, 50%, 67%, and 75%. Please refer to Supplementary Materials Tables S5–S23 for detailed results. To assess the Gaussian distribution of each variable, we conducted the Kolmogorov–Smirnov test (KS test).

#### 2.5.3. Secondary Variables

The variables $x_{i}^{\left(l\right)}$ and $z_{k}^{\left(l\right)}$ underwent a transformation to become binary ordinal variables denoted as follows: ${\tilde{x}}_{i,\alpha }^{\left(l\right)}$ are binary ordinal variables based on the percentiles of $x_{i}^{\left(l\right)}$ variables while ${\tilde{z}}_{k,n}^{\left(l\right)}$ are binary variables defined by various associations of $z_{k}^{\left(l\right)}$ values. Here is how the transformation was performed. Let $x_{i,\alpha }^{\left(l\right)}$ denote the $\alpha^{th}$-percentile of the variable $x_{i}^{\left(l\right)}$, where α takes values from the set {0.25,0.33,0.50,0.67,0.75}. For the case when α=0.50, $x_{i,50}^{\left(l\right)}$ represents the median ($m_{i}^{\left(l\right)}$) of the variable $x_{i}^{\left(l\right)}$. If $x_{i}^{\left(l\right)}$ is less than $x_{i,\alpha }^{\left(l\right)}$, then ${\tilde{x}}_{i,\alpha }^{\left(l\right)}$ is assigned the value 0; otherwise, if $x_{i}^{\left(l\right)}$ is greater than or equal to $x_{i,\alpha }^{\left(l\right)}$, then ${\tilde{x}}_{i,\alpha }^{\left(l\right)}$ is assigned the value 1. As an illustration, consider the variable $x_{i}^{\left(l\right)}$ to be the weight of a patient at the time of diagnosis, belonging to class l=1, with its 0.50 percentile $x_{i,50}^{\left(l\right)}$ being 70 kg. The resulting secondary variable, ${\tilde{x}}_{i,50}^{\left(1\right)}$, takes the value 0 if the patient’s weight is less than 70 kg and 1 if the patient’s weight is greater than or equal to 70 kg. Refer to Tables S5–S23 in the Supplementary Materials for the percentile values corresponding to all the reported variables.

Now, let us consider the variable $z_{k}^{\left(l\right)}$ which has ζ values (where ζ is a positive integer and ζ>2). In this case, n=ζ−1 variables ${\tilde{z}}_{k,n}^{\left(l\right)}$ are obtained by grouping these values. To illustrate how the values of ${\tilde{z}}_{k,n}^{\left(l\right)}$ are derived from the $z_{k}^{\left(l\right)}$ variables, let us suppose $z_{k}^{\left(l\right)}$ represents the pre-treatment (l=3) tumor N-staging with ζ=3 values {N0 or Nx,N1,N2}. As a result, there are n=2 variables ${\tilde{z}}_{k,n}^{\left(l\right)}$ defined as follows: ${\tilde{z}}_{k,1}^{\left(l\right)}$ takes the value 0 if $z_{k}^{\left(l\right)}$ is in the set {N0 or Nx}, and 1 if $z_{k}^{\left(l\right)}$ is in the set {N1,N2}. Similarly, ${\tilde{z}}_{k,2}^{\left(l\right)}$ takes the value 0 if $z_{k}^{\left(l\right)}$ is in the set {N0 or Nx,N1}, and 1 if $z_{k}^{\left(l\right)}$ is in the set {N2}. For the grouping models obtained using this classification method, please refer to Table S3 in the Supplementary Materials.

In order to quantitatively analyze the evolution of hematological variables, all the values of a hematological variable, $X_{h}$ (hemoglobin level, neutrophil count, leukocyte count, platelet count), were recorded at different points of time, $t_{i}$ starting with the one for baseline clinical assessment ($t_{0}$). Based on this string of values, the following extreme values were recorded for the patient: the minimum value reached $X_{h}^{min}=\min{\left\{X_{h}\left(t_{i}\right)\right\}}_{i}$ and maximum variation

$$\Delta X_{h}^{max}={\max\left\{X_{h}\left(t_{0}\right)-X_{h}\left(t_{i}\right)\right\}}_{i}=X_{h}\left(t_{0}\right)-{\min\left\{X_{h}\left(t_{i}\right)\right\}}_{i}$$

of the hematological parameter during intra- and post-therapeutic follow-up. We have also defined the following rates of evolution: maximum rate of change

$$\dot{\Delta }X_{h}^{max}=\frac{\Delta X_{h}^{max}}{t_{i}-t_{0}}$$

and the maximum variation in relation to the total equivalent dose in 2 Gy fractions (EQD2) delivered until the minimum value of the $X_{h}\left(t_{i}\right)$:

$$\Delta_{d}X_{h}^{max}=\frac{\Delta X_{h}^{max}}{{EQD2}_{tot}}$$

The value of ${EQD2}_{tot}$ was computed by adding the EQD2 for EBRT and the EQD2 for brachytherapy, i.e.,

$${EQD2}_{tot}=D_{EBRT}\frac{d+\alpha /\beta }{2+\alpha /\beta }+D_{brachy}\frac{d_{brachy}+\alpha /\beta }{2+\alpha /\beta }$$

where $D_{EBRT}$ and $D_{brachy}$ are the total doses (in Gy) delivered by EBRT and brachytherapy, respectively; d and $d_{brachy}$ are the doses per fraction delivered by EBRT and brachytherapy (typically, $d_{brachy}=7.5\;Gy$), and α/β is a tissue-specific parameter. For cervical cancer, the ratio α/β is typically taken as 10 Gy for acute reactions and 3 Gy for late reactions). We will use 10 Gy in this calculation.

Finally, we have defined a variation rate per number of EBRT fractions, f delivered until the moment when the $X_{h}^{min}$ value has been recorded:

$$\Delta_{f}X_{h}^{max}=\frac{\Delta X_{h}^{max}}{f}$$

#### 2.5.4. Risk Analysis and Logistic Model

Finally, we utilized risk calculations, namely the odds ratio (OR) and risk ratio (RR), to estimate the connections between different categories of categorical binary variables. These categories include variables listed in Table S1 in the Supplementary Materials and the variables ${\tilde{z}}_{k,n}^{\left(l\right)}$. More exactly, we have examined the associations between the treatment outcome variables (in terms of adverse effects) and all possible variables that we investigated and that could be determinants of any of the adverse effects. To determine the statistical significance (*p*-value) of these associations, we employed a logistical univariate model and subsequently a multivariate model. These models linked the outcome primary or secondary binary variables with the independent primary or secondary binary variables.

All statistical analysis was performed using IBM SPSS version 26.

## 3. Results

### 3.1. Patients

A total of 69 patients between the ages of 28 and 76 (with a mean age of 54.19 y, a median age of 53 and a standard deviation of 10.73 y) were included in our study (see Table 2). The majority of our patients (81.0%) did not present any cardiovascular (CV) diseases or high blood pressure at the time of diagnosis. Furthermore, most patients did not have diabetes mellitus nor were they classified as obese (93.1%) when they were diagnosed with cervical cancer, the average body mass index (BMI) of our group being 26.33 kg/m^2^. Only a small percentage of patients (6.9%) presented other types of diseases, including immunity-related conditions.

Other tumor features and detailed staging are presented in Table 3. The histopathological reports of the patients in our group were written by several pathologists, and some of the reports were made in other institutions than our institution where the treatments were performed. Consequently, the reporting was not uniform for all 69 patients. As shown in Table 3, squamous cell carcinoma was the predominant histological type among the patients in our study (94.6%). The most frequently observed histological grade was G2 (58%). The majority of our patients were classified as T2 stage, and over half of them had lymph node involvement at the time of diagnosis (53.4%).

### 3.2. Therapy Modalities

Table 4 shows the combination of treatment modalities in the patients in our group. The majority of our patients underwent treatment consisting of CCRT, followed by brachytherapy. Some of these patients (13 out of a total of 69) did not undergo chemotherapy in our institution and we did not have complete data on chemotherapy. In patients for whom the chemotherapeutic drug was known with certainty, Cisplatin was the most prescribed CHT agent (92.9%) and the RT technique most utilized was 3D-CRT (59.4%). Most patients did not require any breaks during treatment (82.5%). RT has been discontinued (from 1 to 4 weeks) in some patients when adverse reactions of different types (hematological, gastroenterological, genitourinary) have occurred. Among the most difficult to correct side effects that led to interruptions of radiotherapy were moderate thrombocytopenia (between 50,000 and 100,000 per μL) and moderate and severe anemia (hemoglobin levels of 8–10 g/dL and 6.5–8 g/dL). Following CCRT, most of our patients received two brachytherapy fractions (65.2%).

### 3.3. Treatment-Related Adverse Events

The treatment-related adverse events (AE) observed in our patients’ group have been grouped into two categories: clinical AEs and hematological AEs (refer to Table 5). The clinical AEs have been further subdivided into general, genitourinary, and digestive AEs.

Among the clinical AEs, the most frequently reported was fatigue, affecting 63.8% of our patients, while dysuria was the least frequently reported, reported by only 29.3% of our patients. Nausea and vomiting were considered as a single entity, as all patients who experienced nausea (58.6%) also had episodes of vomiting during treatment. Diarrhea occurred in 60.3% of our study group.

### 3.4. Time Period to Maximum Drop of Leukocytes and Hemoglobin

**Figure 2:**
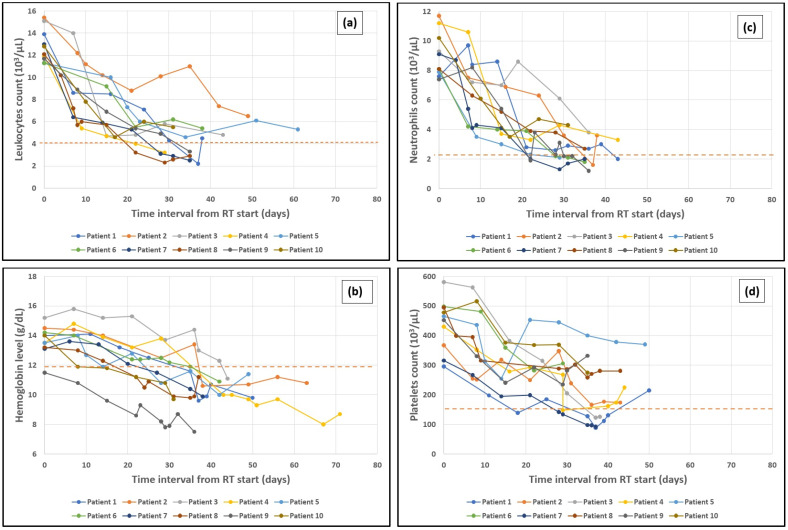
Trends of 4 hematologic variables during intratherapeutic (during radiotherapy) and post-therapeutic monitoring: (**a**) leukocytes, (**b**) hemoglobin, (**c**) neutrophils and (**d**) platelets in 4 different sets of 10 randomly selected patients from our cohort. The dashed orange lines represent the lower limits of the range of normal reference values of our institution’s laboratory for (**a**) leukocytes (4 × 10^3/^μL), (**b**) hemoglobin (12 g/dL), (**c**) neutrophils (2.2 × 10^3/^μL) and (**d**) platelets (150 × 10^3/^μL).

For this section, we have defined ‘time period’ as the duration (measured in days) between baseline blood test values and the point during treatment when the lowest values were reached. In Figure 2, we have plotted the time evolutions of four hematologic variables (together with the lower limit of the reference value ranges for these variables) during radiotherapy and post-therapeutic monitoring: (a) leukocytes, (b) hemoglobin, (c) neutrophils, and (d) platelets in four different sets (one for each variable) of 10 randomly selected patients from our cohort. The general trend of these variables being a downward one, we explored the existence of risk factors for lowering their values towards limits that would hinder the treatment course and the patients’ quality of life.

The maximum drop in leukocytes was observed before day 35 from the RT initiation (refer to Table 6 but also to Figure 2a for illustration) in patients who underwent treatment with 3D-CRT (OR = 4.44, 95%CI = [1.25, 15.82], *p* = 0.021, RR = 1.94, 95%CI = [1.00, 3.78]). Day 35 falls outside the timeframe in which EBRT was administered, usually coinciding with the time interval just before the first brachytherapy application. This drop in leukocytes on the above-mentioned time interval since the initial blood tests were taken is associated with a total planned RT dose and a pelvic volume RT dose of over 50.4 Gy which in turn corresponds to a fractionation regimen of more than 28 fractions. Similarly, this association holds true for the total planned dose delivered by EBRT and brachytherapy, with a total EQD2 over 66.1 Gy in patients with CTV that includes lumboaortic lymph nodes (OR = 4.00, 95%CI = [1.19, 13.50], *p* = 0.025, RR = 1.75 95CI = [1.02, 3.00]).

Hemoglobin also showed a quantifiable decreasing trend (see Figure 2b) as EBRT progressed. Regarding the time period between the baseline hemoglobin and the maximum hemoglobin drop, we observed that these low hemoglobin values were reported after day 29 and a total EQD2 over 66.1 Gy (EBRT and brachytherapy). However, this is more likely to occur after day 35, around the time of the first brachytherapy application. There is also a strong association between the maximum hemoglobin drop after day 35 and the RT technique, with a greater impact on patients treated with 3D-CRT as opposed to VMAT.

### 3.5. Neutrophils and Platelets Drop

Both neutrophil and platelet counts decrease (see Figure 2c,d) in a dose-dependent manner (refer to Table 6). Neutrophils count below 1.82 × 10^3^/μL and platelet count below 131 × 10^3^/μL were found to be correlated with a pelvic region RT dose of 54 Gy.

Neutropenia is more likely to occur in patients whose rectal V45 and bladder V50 exceeded 63.43% and 10.69%, respectively. Moreover, the occurrence of neutropenia was strongly associated with a bowel V45 of over 137.38 cc.

### 3.6. Hemoglobin Drop over the Course of Treatment

A maximum drop in hemoglobin of over 2.30 g/dL was observed in patients aged over 53 and was also found to be associated with a BMI of over 23.23 kg/m^2^ (refer to Table 6). Additionally, a drop of over 2.50 g/dL occurred more frequently in patients treated with 3D-CRT as opposed to VMAT (OR = 7.25, 95%CI = [1.73, 30.38], *p* = 0.007, RR = 4.13, 95% CI = [2.63, 6.49]). This maximum drop was more commonly recorded in patients who received a total EQD2 dose that exceeded 66.1 Gy and the CTV included lomboaortic lymph nodes.

The rate of hemoglobin variation over time is RT dose-dependent (refer to Table 7). A variation exceeding 0.03 g/dL/day was associated with a total planned RT dose, including EBRT and brachytherapy, a total EQD2 dose that exceeded 66.1 Gy, along with a planned pelvic volume and lomboaortic lymph nodes dose of over 50.4 Gy.

A hemoglobin variation rate per dose of over 0.04 g/dL/Gy had a strong correlation with the pelvic region dose, as well as the total planned dose across all volumes exceeding 50.4 Gy. This equates to a treatment plan consisting of more than 28 fractions. The total EQD2 of over 66.1 Gy also impacted the dose-dependent hemoglobin variation rate. The same factors are associated with a per fraction hemoglobin variation rate of over 0.08 g/dL/fraction.

## 4. Discussion

### 4.1. Hematological Adverse Events

The main objective was to determine associations between the occurrence of these adverse effects and clinical variables related to patients, as well as variables related to the modes of radio-treatment. Such associations would be very useful in constructing risk stratification models for the occurrence of these effects in patients, thus allowing for the implementation of preventive and prophylactic measures.

Anemia was detected in 77.9% of the patients in our study, which is comparable to the findings of a prospective study assessing acute post-RT adverse events in patients treated with CCRT, where anemia was observed in 69.2% of patients [18]. Anemia, tumor hypoxia, and increased angiogenesis have been associated with a poor prognosis and lower OS and progression-free survival (PFS) rates [19,20]. Furthermore, in our study group, decreases of more than 2.3 g/dL in the hemoglobin level are more likely in patients with BMI > 23 kg/m^2^ and with an age over 53 years and are also associated with 3D-CRT. The hemoglobin level during treatment serves as a reliable predictor for local control and survival. The association of greater hemoglobin drops with 3D-CRT is in agreement with studies that demonstrated IMRT ability to minimize bone marrow tissue exposure without compromising adequate target coverage [21]. Certain studies indicate the superiority of VMAT over IMRT in terms of the incidence rate of acute anemia [14]. Beyond the obvious indication to choose VMAT over IMRT, our results would indicate that, in institutions where 3D-CRT has not yet been completely replaced, there is a need for closer monitoring of patients with BMI > 23 kg/m^2^ or older than 53 years old who have a hemoglobin level around 14 g/dL at the beginning of radiotherapy.

The most likely rates (expressed in various ways) of decrease in hemoglobin according to our study (see Table 7) start with 0.03 g/dL/day, 0.04 g/dL/Gy and 0.08 g/dL/fraction. Consider this 9 g/dL hemoglobin value as the critical cutoff for patients to continue RT. For a course of EBRT in 28 fractions (5 fractions/week) the total drop in hemoglobin at the end of the course would be 1.1 g/dL which means that patients, without other risks of anemia with a hemoglobin of over 10.1 g/dL at the beginning of the therapy could follow such a cure without the need for a transfusion. Risk factors would double this irradiation so that patients who would present such risk factors should have a hemoglobin level of at least 11.2 g/dL at the beginning of the treatment to tolerate EBRT well without needing transfusions. Transfusions have been largely utilized to treat anemia in patients undergoing RT. An expert consensus guideline for packed red blood cell transfusion protocols recommends a target minimum hemoglobin value of 9 g/dL for cervical cancer patients receiving EBRT and brachytherapy, though maximization of clinical benefits has yet to be achieved and further trials are required [22]. EQD2 values > 66.1 Gy or lumboortic lymph node irradiation are also associated with the largest decreases in hemoglobin (absolute or normed in time, dose or fraction).

Leukopenia is a common hematological AE observed in 66.2% of our patients undergoing CCRT observed with greater probability before 35 days after RT start with a greater risk when 3D-CRT was used. As in the case of hemoglobin, this maximum decrease is related to the 3D-CRT technique and the above recommendations stand, but this time the concerned patients have those who have levels closer to the limit value, 4 × 10^3^/μL. For instance, tomotherapy was shown to induce leukopenia with a lower frequency than IMRT [23]. A comparison of definitive RT and CCRT in an elderly patient population revealed that RT alone can serve a viable treatment alternative, posing no significant differences to CCRT in terms of complete response, OS and PFS. Moreover, definitive RT was associated with a lower occurrence of severe leukopenia compared to CCRT [24].

According to our results the maximum drop in leukocytes count is more probable when EQD2 > 66.1 Gy and the lomboaortic lymph nodes are included in the irradiation volume. This drop might be explained by the irradiation of the wide bones with a role in hematopoiesis. Hence, dosimetric parameters and bone marrow delineation can significantly affect the incidence of acute and chronic HTs. A study demonstrated that contouring the inner bone cavity on CT images had a stronger correlation with high-grade HT compared to contouring the entire bone, suggesting that the inner bone cavity serves as a superior surrogate for bone marrow [25]. To reduce the risk of severe HT, dose constraints can be implemented, such as maintaining the total pelvic bone V20 below 65% and a mean dose under 31 Gy for the iliac crests, as proposed by Kumar et al. in their 2019 study [26]. Furthermore, limiting bone marrow V10 and V20 can be beneficial in mitigating HTs like leukopenia and neutropenia [27]. The association of these EQD2 values > 66.1 Gy with decreases in hemoglobin levels and leukocytes counts indicates that the moment of transition to brachytherapy and the peri-brachytherapy period seem to be critical for patients who start radiotherapy with low hematological indicators: elderly patients, in an advanced stage of the disease, with malabsorptions, etc. Our results indicate this moment as being within a given interval of the moment of the maximum variation of hemoglobin and that of the maximum variation of leukocytes count, i.e., between 29 and 36 days from RT initiation. Low white blood cell counts as well as anemia in patients receiving CCRT can lead to discontinuation or delay of treatment.

Neutropenia and febrile neutropenia can often occur in patients receiving CCRT. Although no cases of febrile neutropenia have been observed in our study, 42.6% of our patients experienced low neutrophil counts during the treatment period, with neutrophils level drop of over 1.82 × 10^3^/μL (see Table 6) with respect to the pre-treatment neutrophils level. The lower value of the reference interval is different in each institution and this variation allows the calculation of an indicative red flag value from which patients cannot undergo EBRT treatment. In our institution, the minimum threshold value is 2.2 × 10^3^/μL, so patients with neutrophil count values below 4 × 10^3^/μL are at risk of neutropenia. Obviously, as in the example of anemia, this calculation is sophisticated enough to be applied according to risk factors, since this variation in the level of neutrophils does not take into account other clinical variables (for example, age, hematological history, etc.) whose values would be indicators of non-negligible risks of neutropenia. As a preventive measure for neutropenia, we mention the pegylated recombinant human granulocyte colony-stimulating factor, which has been proven to be both safe and effective in preventing severe neutropenia over the course of CCRT. Additionally, the treatment group demonstrated lower incidences of febrile neutropenia and reduced time delays in CHT cycles compared to the control group [3].

Although no associations of the onset of neutropenia with marrow irradiation values were obtained, as long as the dose constraints related to this OAR were respected in the plan validation stage, neutropenia in the patients in our study was observed in the patients for whom rectal V45 and bladder V50 in VMAT planning were over 63.4% and 10.6%, respectively. This result shows that although the dose constraints to the hematogenous marrow can be respected, the two cited parameters can be considered as predictors of the risk of hematogenous AEs as a result of global pelvis irradiation.

### 4.2. Clinical Adverse Events

In our study, we did not find associations between general, genitourinary, and digestive symptoms and the variables we monitored. Although our patients have experienced clinical AEs, these have been proven to be independent of the other variables in our study.

Fatigue, as a general AE, has been reported by 53.6% of our patients during treatment, and this finding is consistent with other studies. such as Aishanjiang et al. who reported fatigue as a prevalent symptom in two groups submitted to EBRT [28].

Chronic fatigue can persist in the long-term post-RT, with self-reported fatigue scores over a 5-year period being higher in cervical cancer survivors compared to the general population. This finding underlines the clinical importance of this symptom and its impact on patients’ quality of life (QoL), not only during treatment, but also in the follow-up period [11].

Dysuria was observed in 17 patients within our study group, accounting for 29.3% of the participants. This percentage is significantly higher compared to that reported in a study by Kibaara and Degu which aimed to assess the prevalence of AEs in patients undergoing CCRT. The two most encountered AEs in relation to RT were ulcerated sores (52.8%) and dysuria (7.5%) [29]. Pelvic irradiation affects various healthy structures and organs, including the intestines and bladder. These organs are susceptible to both early and late toxicity associated with the damaging effects of ionizing radiation, both during and after irradiation. Numerous studies have found that approximately 50% of patients undergoing pelvic irradiation experience radiation toxicity during treatment [30]. Regarding digestive AEs, cervical cancer patients most frequently reported experiencing nausea, vomiting, and diarrhea. Our study results indicate that nausea and vomiting only occurred together, although that is not always the case. A study that aimed to evaluate the non-inferiority of two different CCRT treatment regimens, one with weekly nedaplatin and the other with weekly cisplatin, revealed that nausea and vomiting were more prevalent in the cisplatin group, whereas nedaplatin posed a higher risk of hepatotoxicity. The 3-year OS in both groups was similar, suggesting that nedaplatin could be a viable alternative to cisplatin in the treatment of cervical cancer [31].

On the other hand, nausea and vomiting can be evaluated as purely RT-induced AEs, as depicted in a study conducted by Izmajłowicz et al. Approximately three-quarters of the patients experienced nausea, while vomiting was reported by 20.9% [18]. In comparison to our findings, nausea was more prevalent, whereas vomiting occurred less frequently in our group of patients.

### 4.3. Study Limitations

Our study has some limitations. For instance, systematic time series of hematological data time evolution were not obtained for all patients. Our study was a prospective one and it was not conducted within a formal adverse reaction monitoring program, and, overall, the hematological parameters of our patients were not monitored regularly and frequently, venous blood collection not being a procedure to which patients readily adhere.

To this, the non-availability of histopathological reports was added for some patients because the pathological study of the tumor was performed in another institution either because the surgical intervention or the biopsy was performed elsewhere than in the institution where the radiotherapy was performed. The non-uniformity in the reporting of tumor features caused either by the use of resection pieces or biopsy pieces in the pathological study (the latter limiting the extent of detail in tumor features descriptions by the amount of tissue) or by the writing of these reports by different pathologists with different experiences and styles. It should be noted that both at the institutional and at the national level there are pathological reporting templates.

Secondly, not all patients were administered with all treatment modalities in our institution. Although harmful for intratherapeutic and post-therapeutic patient follow-up in the absence of a digital patient medical record, this is allowed by the territorial regulations of medical services. For this reason, complete chemotherapy data were not available in some patients.

Thirdly, due to the small sample size, it was not possible to build multivariate predictive models. Finally, the long-term effects, especially on quality of life and fertility (especially in the case of low-grade cancers), have not been monitored. Fertility is an essential aspect for gynecological cancer patients. In patients with early-stage gynecologic malignancies, fertility-sparing approaches like hormonal therapy or hysteroscopic tumor resection followed by hormonal therapy have proven to give good results from both an oncological and reproductive standpoint [32,33]. Progesterone receptor was found to be particularly promising in predicting positive responses, though no reliable predictive markers are readily available for clinical use [34].

## 5. Conclusions

In this paper, we investigated the adverse effects of radiochemotherapy in patients with cervical cancer, focusing on the AR in the peri-RT interval. Within our study, we were able to quantify the risks of the hematological adverse effects: an EQD2 > 66.1 Gy, pelvic irradiation associated with lumboaortic lymph node irradiation as using the 3D-CRT technique represent risk factors for decreased hemoglobin (reported in different forms: absolute or time-normed, dose or fraction) and leukocytes count. Our calculations show the existence of a transition interval located somewhere between 29 and 35 days from the moment of EBRT initiation, in which the two variables can reach critical values, especially in patients with a precarious clinical status and low hematological values. For example, patients in this situation over 53 years of age and BMI > 23.23 kg/m^2^ should be closely monitored during the break between EBRT and brachytherapy. Our study opens up perspectives for systematic quantitative evaluations of adverse effects on larger batches with more complex models than the linear regression ones we used.

## Figures and Tables

**Table 1:** Classes of variables used in this study.

l	Data Class
1	Clinical and Demographical
2	Pre-treatment tumor pathology features
3	Treatment modalities
4	Radiotherapy-related adverse events
5	Post-treatment characteristics

**Table 2:** Clinical and demographic characteristics, comorbidities, and their presence in the patients group.

Variable		Age (in yrs)	Weight (in kg)	BMI (in kg/m^2^)
Mean		54.19	69.47	26.33
Range		48	62	20.06
Minimum		28	40	15.63
Maximum		76	102	35.69
Percentiles	0.25	48.50	61.00	23.23
	0.33	50.14	63.28	24.64
	0.50	53.00	70.00	26.56
	0.67	59.00	75.00	27.86
	0.75	62.50	76.00	28.90
**Variable**		**Value**	**N**	**%**
High blood pressure and/or other CV diseases		Yes	11	15.9
		No	58	84.0
DM and/or obesity		Yes	4	5.7
		No	65	94.2
CV disease and DM or obesity combined		Yes	10	14.4
		No	59	85.5
Other diseases or immunity-related		Yes	4	5.7
		No	65	94.2

CV–cardiovascular; DM—diabetes mellitus.

**Table 3:** Tumor histology and staging.

Characteristic	Value	N	%
Histopathological type	Squamous cell carcinoma	53	94.6
	Adenocarcinoma	3	5.4
Histopathological differentiation	G1	1	2.4
	G2	24	58.5
	G3	16	39.0
Presence of invaded lymph nodes	No	27	46.6
	Yes	31	53.4
Pre-treatment tumor (T) staging	T1a2	4	7.4
	T1b	2	3.7
	T1b1	1	1.9
	T1b2	1	1.9
	T1b3	1	1.9
	T2a	36	66.7
	T2a2	6	11.1
	T2b	3	5.6
Pre-treatment nodal status (N)	Nx or N0	27	46.6
	N1	26	44.8
	N2	5	8.6
Pre-treatment AJCC Staging	Stage IB1	3	5.5
	Stage IB2	1	1.8
	Stage IIB	2	3.6
	Stage III	20	36.4
	Stage IIIC1	7	12.7
	Stage IIIC2	17	30.9
	Stage IVA	3	5.5
	Stage IVB	2	3.6

**Table 4:** Treatment modalities employed and subsequent delays in treatment.

Characteristic	Value	N	%
Chemotherapy	No chemotherapy	1	1.8
	Cisplatin	52	92.9
	Carboplatin	3	5.4
	NA	13	
Radiotherapy technique	3D-CRT	41	59.4
	VMAT	18	26.1
Brachytherapy fractions	0	4	5.8
	2	45	65.2
	3	9	13.0
	4	1	1.4
Duration (in weeks) of radiotherapy breaks	0	49	82.5
	1	5	8.8
	2	4	7.0
	4	1	1.8

NA—not available.

**Table 5:** Treatment-related adverse reactions.

Characteristic	Value	N	%
**General**			
Fatigue	No	21	36.2
	Yes	37	63.8
	Unknown	10	
**Genitourinary**			
Dysuria	No	41	70.7
	Yes	17	29.3
	Unknown	10	
**Digestive**			
Nausea and vomiting	No	24	41.4
	Yes	34	58.6
	Unknown	10	
Diarrhea	No	23	39.7
	Yes	35	60.3
	Unknown	10	
**Hematological**			
Anemia	No	15	22.1
	Yes	53	77.9
Thrombocytopenia	No	43	63.2
	Yes	25	36.8
Neutropenia	No	39	57.4
	Yes	29	42.6
Leukopenia	No	23	33.8
	Yes	45	66.2

**Table 6:** Associations between radiation planned doses and hematological adverse effects and quantitative parameters.

Variable	OR	95%CI	*p*	RR	95%CI
**Neutropenia with a minimal neutrophils level ≤ 2.2; (VN: 2.0–7.7) (in 10^3^/μL) during follow-up**					
Planned dose for pelvic region volume = 54 Gy	6.82	(1.15, 40.41)	0.034	2.56	(1.53, 4.29)
**Thrombocytopenia with a minimal platelets level ≤ 131.00; (VN: 150–400) (in 10^3^/μL) during follow-up**					
Planned dose for pelvic region volume = 54 Gy	6.67	(1.22, 36.59)	0.029	1.95	(1.34, 2.83)
**Neutropenia during follow-up**					
Rectal V45 as planned within VMAT > 63.43%	18.00	(1.19, 271.46)	0.037	5.26	(2.86, 9.67)
Bladder V50 as planned within VMAT > 10.69%	16.50	(1.09, 250.18)	0.043	4.88	(2.66, 8.93)
Bowel V45 as planned within VMAT > 137.38 cc	16.50	(1.09, 250.18)	0.043	4.88	(2.66, 8.93)
**Maximum drop of leukocytes level in less than 35 days from RT initiation**					
Radiotherapy technique = 3D-CRT	4.44	(1.25, 15.82)	0.021	1.94	(1.00, 3.78)
Total EQD2 > 66.1 Gy(CTV includes lomboaortic lymph nodes)	4.12	(1.20, 14.14)	0.025	1.75	(1.04, 2.93)
Planned dose for pelvic region volume = 54 Gy	4.00	(1.19, 13.50)	0.025	1.75	(1.02, 3.00)

**Table 7:** Associations between hemoglobin levels and their variations and BMI, age and other RT planning related variables.

Variable	OR	95%CI	*p*	RR	95%CI
**Maximum Hb variation > 2.50 g/dL**					
BMI > 23.23 kg/m^2^	8.68	(1.01, 75.01)	0.049	5.29	(2.31, 12.14)
Age > 53 yrs	4.60	(1.10, 19.22)	0.036	2.99	(1.84, 4.86)
Radiotherapy technique 3D-CRT	7.25	(1.73, 30.38)	0.007	4.13	(2.63, 6.49)
Total EQD2 > 66.1 Gy(CTV includes lomboaortic lymph nodes)	4.71	(1.09, 20.47)	0.038	3.26	(1.95, 5.44)
**Per day maximum Hb variation rate > 0.03 g/dL/day**					
Total EQD2 > 66.1 Gy(CTV includes lomboaortic lymph nodes)	11.81	(1.35, 103.04)	0.025	1.49	(1.31, 1.71)
Planned dose for pelvic region volume = 54 Gy	10.56	(1.22, 91.01)	0.032	1.48	(1.30, 1.68)
**Per dose maximum Hb variation rate > 0.04 g/dL/Gy**					
Total EQD2 > 66.1 Gy(CTV includes lomboaortic lymph nodes)	10.50	(2.00, 55.03)	0.005	1.82	(1.52, 2.19)
Planned dose for pelvic region volume = 54 Gy	8.82	(1.71, 45.52)	0.009	1.75	(1.47, 2.07)
Planned radiotherapy fractions > 28.00 fractions	8.82	(1.71, 45.52)	0.009	1.75	(1.47, 2.07)
**Per dose maximum Hb variation rate > 0.08 g/dL/fraction**					
Total EQD2 > 66.1 Gy(CTV includes lomboaortic lymph nodes)	8.89	(1.69, 46.63)	0.01	1.69	(1.42, 2.00)
Planned dose for pelvic region volume = 54 Gy	7.60	(1.47, 39.29)	0.016	1.63	(1.39, 1.91)
**Maximum Hb variation in less than 29 days from RT initiation**					
Planned dose for pelvic region volume = 54 Gy	6.76	(1.88, 24.29)	0.003	2.85	(1.27, 6.38)
Total EQD2 > 66.1 Gy(CTV includes lomboaortic lymph nodes)	6.02	(1.72, 21.10)	0.005	2.61	(1.24, 5.46)
**Maximum Hb variation in less than 35 days from the RT initiation**					
Planned dose for pelvic region volume > 50.40 Gy	11.11	(2.54, 48.66)	0.001	5.32	(3.25, 8.71)
Total EQD2 > 66.1 Gy(CTV includes lomboaortic lymph nodes)	8.00	(1.86, 34.36)	0.005	4.35	(2.66, 7.11)
Radiotherapy technique (1—VMAT, 2—3D-CRT)	3.71	(1.03, 13.46)	0.035	2.36	(1.65, 3.37)

## Data Availability

The data presented in this study are available in the present paper.

## Supplementary Materials

The following supporting information can be downloaded at: https://www.mdpi.com/article/10.3390/cancers16173032/s1, Table S1 Primary qualitative variable and their values; Table S2 Categories and subcategories of primary quantitative variables; Table S3 List of binary ordinal variables defined by grouping the values of multinomial variables; Table S4 Variation rates of blood counts; Table S5 Calculated mean (and its standard error), standard deviation, variance, extreme values (minimum and maximum) and the 25th, 33rd, 50th, 67th and 75th percentiles of age, weight, BMI for the patients in the study group; Table S6 Calculated mean (and its standard error), standard deviation, variance, extreme values (minimum and maximum) and the 25th, 33rd, 50th, 67th and 75th percentiles of the variables related to the radiotherapy treatment planning and delivery for the patients in the study group; Table S7 Calculated mean (and its standard error), standard deviation, variance, extreme values (minimum and maximum) and the 25th, 33rd, 50th, 67th and 75th percentiles of the baseline and minimum values recorded during treatment within this study group for hemoglobin, platelets, neutrophils, and leukocytes; Table S8 Calculated mean (and its standard error), standard deviation, variance, extreme values (minimum and maximum) and the 25th, 33rd, 50th, 67th and 75th percentiles of the hemoglobin variation rates for the patients in the study group; Table S9 Calculated mean (and its standard error), standard deviation, variance, extreme values (minimum and maximum) and the 25th, 33rd, 50th, 67th and 75th percentiles of the platelets variation rates. Normal values interval 150–400×103/μL; Table S10 Calculated mean (and its standard error), standard deviation, variance, extreme values (minimum and maximum) and the 25th, 33rd, 50th, 67th and 75th percentiles of the neutrophils variation rates. Normal values interval 2.2–7.7×103/μL; Table S11 Calculated mean (and its standard error), standard deviation, variance, extreme values (minimum and maximum) and the 25th, 33rd, 50th, 67th and 75th percentiles of the leukocytes variation rates. Normal values interval 4–11×103/μL; Table S12 Calculated mean (and its standard error), standard deviation, variance, extreme values (minimum and maximum) and the 25th, 33rd, 50th, 67th and 75th percentiles of the fractions (F) and doses (D) in Gy delivered up to blood test minimum values recorded during treatment; Table S13 Calculated mean (and its standard error), standard deviation, variance, extreme values (minimum and maximum) and the 25th, 33rd, 50th, 67th and 75th percentiles of the pre-treatment and post-treatment MRI tumor volumes; Table S14 Calculated mean (and its standard error), standard deviation, variance, extreme values (minimum and maximum) and the 25th, 33rd, 50th, 67th and 75th percentiles of the rectal V40, V45, V50 (in VMAT and 3D); Table S15 Calculated mean (and its standard error), standard deviation, variance, extreme values (minimum and maximum) and the 25th, 33rd, 50th, 67th and 75th percentiles of the bladder V40, V45, V50 (in VMAT and 3D); Table S16 Calculated mean (and its standard error), standard deviation, variance, extreme values (minimum and maximum) and the 25th, 33rd, 50th, 67th and 75th percentiles of the bowel V45 (in %) in VMAT and 3D; Table S17 Calculated mean (and its standard error), standard deviation, variance, extreme values (minimum and maximum) and the 25th, 33rd, 50th, 67th and 75th percentiles of the right and left kidney V18 (in VMAT and 3D); Table S18 Calculated mean (and its standard error), standard deviation, variance, extreme values (minimum and maximum) and the 25th, 33rd, 50th, 67th and 75th percentiles of the mean and maximum dose to be delivered to the right and left kidneys (in VMAT and 3D); Table S19 Calculated mean (and its standard error), standard deviation, variance, extreme values (minimum and maximum) and the 25th, 33rd, 50th, 67th and 75th percentiles of the right and left femoral heads V45 and V50 (in VMAT and 3D); Table S20 Calculated mean (and its standard error), standard deviation, variance, extreme values (minimum and maximum) and the 25th, 33rd, 50th, 67th and 75th percentiles of the maximum doses to be delivered to the left and right femoral heads (in VMAT and 3D); Table S21 Calculated mean (and its standard error), standard deviation, variance, extreme values (minimum and maximum) and the 25th, 33rd, 50th, 67th and 75th percentiles of the hematogenous marrow V30 and V40 (in VMAT and 3D); Table S22 Calculated mean (and its standard error), standard deviation, variance, extreme values (minimum and maximum) and the 25th, 33rd, 50th, 67th and 75th percentiles of the maximum doses to be delivered to the hematogenous marrow (in VMAT and 3D); Table S23 Calculated mean (and its standard error), standard deviation, variance, extreme values (minimum and maximum) and the 25th, 33rd, 50th, 67th and 75th percentiles of the 95% isodose (in VMAT and 3D).

## Abbreviation

3D-CRT	3D-conformal radiotherapy
AE	Adverse event
BMI	Body mass index
CCRT	Concurrent chemoradiotherapy
CHT	Chemotherapy
CT	Computed tomography
CTV	Clinical target volume
CV	Cardiovascular
DM	Diabetes mellitus
DVH	Dose-volume histogram
EBRT	External beam radiotherapy
EF	Extended field
GTV	Gross tumor volume
HT	Hematological toxicity
IMRT	Intensity-modulated radiotherapy
OAR	Organ at risk
OR	Odds-ratio
OS	Overall survival
PFS	Progression-free survival
PTV	Planning target volume
QoL	Quality of life
RT	Radiotherapy
RR	Risk-ratio
UAIC	Uterine arterial interventional chemoembolization
VMAT	Volumetric modulated arc therapy
