# Supplementary material for: Predictors of Clinical Hematological Toxicities under Radiotherapy in Patients with Cervical Cancer—A Risk Analysis
Source: Cancers (Basel). 2024 Aug 30;16(17):3032. doi: 10.3390/cancers16173032 (PMC11394146; doi:10.3390/cancers16173032)
Supplement: Supplementary file 1 [file cancers-16-03032-s001.zip › cancers-3091228-supplementary.pdf]

Predictors of Clinical Hematological Toxicities under Radiotherapy in Patients with Cervical Cancer—A Risk Analysis

Șerban Andrei Marinescu [1,†], Radu-Valeriu Toma [1,2,†], Oana Gabriela Trifănescu [1,2], Laurenția Nicoleta Galeș [1,2], Antonia Ruxandra Folea [1,2], Adrian Sima [3], Liviu Bîlteanu [2,4,*] and Rodica Anghel [2]

1 Oncological Institute "Alexandru Trestioreanu" Bucharest, 252 Soseaua Fundeni, 022328 Bucharest, Romania; serban.marinescu@yahoo.com (Ș.A.M.); radu.toma@umfcd.ro (R.-V.T.); oana.trifanescu@umfcd.ro (O.G.T.); laurentia.gales@umfcd.ro (L.N.G.); antonia.folea@gmail.com (A.R.F.)

2 Faculty of General Medicine, Carol Davila University of Medicine and Pharmacy, 8 Eroilor Sanitari Street, 050474 Bucharest, Romania; rodicamanghel@gmail.com

3 Department of Mathematics, Physics and Terrestrial Measurements, Faculty of Land Improvements and Environmental Engineering, University of Agronomic Sciences and Veterinary Medicine, 105 Splaiul Independentei, 050097 Bucharest, Romania; adriansima1981@gmail.com

4 Laboratory of Molecular Nanotechnologies, National Institute for Research and Development in Microtechnologies, 126A Erou Iancu Nicolae Street, 077190 Voluntari, Romania

* Correspondence: liviu.bilteanu@imt.ro; Tel.: +40-21-318-04-69

Supplementary Materials

Table S1 Primary qualitative variable and their values

**Clinico-demographics**	
Address	1 – rural<br>2 – urban
**Pre-treatment characteristics**	
Histopathological Type	1 – squamous<br>2 – adenocarcinoma
Histopathological Differentiation	1 – G1<br>2 – G2<br>3 – G3
p16 Mutation Presence	1 – yes<br>0 – no
Comorbidities	1 – HTA and/or other CV diseases<br>2 – diabetes and/or obesity<br>3 – CV disease and DZ or obesity combined<br>4 – other diseases or immunity-related
Pre-treatment Staging	Stages according to AJCC 8th edition
Pre-treatment T Staging	T Stages according to AJCC 8th edition
Pre-treatment N Staging	0 – Nx or N0<br>1 – N1<br>2 – N2

Presence of invaded lymph nodes	1 – yes<br>0 – no
Vaginal invasion	1 – yes<br>0 – no
Vaginal invasion up to the distal 1/3 segment	1 – yes<br>0 – no
Vaginal invasion beyond the distal 1/3 segment	1 – yes<br>0 – no
Was a pre-therapeutic MRI performed?	1 – yes<br>0 – no
**Treatment modalities**	
Chemotherapy course	1 – cisplatin<br>2 – carboplatin<br>3 – other
Radiotherapy technique	1 – 3D<br>2 – VMAT
Radiotherapy target volume	1 – pelvis only<br>2 – pelvis and inguinal lymph nodes<br>3 – pelvis and lombo-aortic lymph nodes<br>4 – pelvis, inguinal and lombo-aortic lymph nodes
Was a boost dose administered?	1 – yes<br>0 – no
**Radiotherapy-related adverse events**	
Nausea	1 – yes<br>0 – no
Vomiting	1 – yes<br>0 – no
Diarrhea	1 – yes<br>0 – no
Dysuria	1 – yes<br>0 – no
Fatigue	1 – yes<br>0 – no
Anemia	1 – yes<br>0 – no
Thrombocytopenia	1 – yes<br>0 – no
Neutropenia	1 – yes<br>0 – no
Leukopenia	1 – yes<br>0 – no
**Post-treatment characteristics**	
Treatment response	1 – complete<br>0 – partial
Did the patient undergo surgery?	1 – yes<br>0 – no
Post-treatment histopathological phenotypes	1 – squamous carcinoma<br>2 – adenocarcinoma<br>0 – complete response
Post-treatment Staging	Stages according to AJCC 8th edition
Post-treatment T Staging	T Stages according to AJCC 8th edition

Post-treatment N Staging	0 – Nx or N0<br>1 – N1<br>2 – N2
Was the post-therapeutic progression of the disease recorded?	1 – yes<br>0 – no

Tumor staging according to the following reference:

Amin, M. B., Edge, S. B., Greene, F. L., Schilsky, R. L., Brookland, R. K., Washington, M. K., Gershenwald, J. E., Compton, C. C., Hess, K. R., Sullivan, D. C., Jessup, M. J., Brierley, J. D., Gaspar, L. E., Balch, C. M., Winchester, D. P., Asare, E. A., Madera, M., Gress, D. M.; Meyer, L. R. (2017). American Joint Committee on Cancer (AJCC), AJCC Cancer Staging Manual, (8th edition).

Table S2 Categories and subcategories of primary quantitative variables

**Baseline patient data**	Age (in yrs)<br>Weight (in kg)<br>Height (in m)<br>BMI (in kg/m^2)	
**Pre-treatment characteristics**	**Pathological features**	Ki67 Level (in %)<br>Number of invaded lymph nodes in pelvis<br>Number of invaded lymph nodes in lombo-aortic area<br>Total number of invaded lymph nodes
	**MRI tumor volumes**	Pre-treatment DWI tumor volume (cc)<br>Pre-treatment T2 tumor volume (cc)
**Post-treatment characteristics**	**MRI tumor volumes**	Post-treatment DWI tumor volume (cc)<br>Post-treatment T2 tumor volume (cc)
**Blood count**	**Hemoglobin**	Initial hemoglobin level (in g/dl; VN: 12-18)<br>Minimal hemoglobin level (in g/dl; VN: 12-18)<br>Fractions delivered up to the moment when minimal hemoglobin is measured<br>Dose (in Gy) delivered up to the moment when minimal hemoglobin is measured<br>Time period (in days) in between the initial value and the maximum drop of hemoglobin
	**Platelets**	Initial platelets level (in 10^3/µl; VN: 150-400)<br>Minimal platelets level (in 10^3/µl; VN: 150-400)<br>Fractions delivered up to the moment when minimal platelets are measured<br>Dose (in Gy) delivered up to the moment when minimal platelets are measured<br>Time period (in days) in between the initial value and the maximum drop of platelets
	**Neutrophils**	Initial neutrophils level (in 10^3/µl; VN: 2.0-7.7)<br>Minimal neutrophils level (in 10^3/µl; VN: 2.0-7.7)<br>Fractions delivered up to the moment when minimal neutrophils are measured<br>Dose (in Gy) delivered up to the moment when minimal neutrophils are measured<br>Time period (in days) in between the initial value and the maximum drop of neutrophils
	**Leukocytes**	Initial leukocytes level (in 10^3/µl; VN: 4-11)<br>Minimal leukocytes level (in 10^3/µl; VN: 4-11)<br>Fractions delivered up to the moment when minimal leukocytes are measured<br>Dose (in Gy) delivered up to the moment when minimal leukocytes are measured

		Time period (in days) in between the initial value and the maximum drop of leukocytes
**Radiotherapy**	**Treatment planning and delivery**	Planned dose for pelvic region volume (in Gy)<br>Dose fraction (in Gy/fraction)<br>Planned radiotherapy fractions<br>Planned dose for lombo-aortic lymph nodes volume (in Gy)<br>Planned boost dose on lymph nodes (in Gy)<br>Total planned dose in all volumes (in Gy)<br>Number of brachytherapy fractions<br>Total planned brachytherapy dose (in Gy)<br>Total EQD2 dose by external irradiation and brachytherapy (in Gy)<br>Cover (in %) 95%<br>Duration (in weeks) of radiotherapy halt
	**OAR Volumes (both 3D and VMAT)**	Rectal V40, V45, V50,<br>Bladder V40, V45, V50<br>Bowel V45<br>Right and left kidney V18, mean dose, maximum dose<br>Right and left femoral heads V45, V50, maximum dose<br>Hematogenous marrow V30, V40, maximum dose

Table S3 List of binary ordinal variables defined by grouping the values of multinomial variables

Histopathological Differentiation Model 1	1 - G1<br>2 - G2 or G3
Histopathological Differentiation Model 2	1 - G1 or G2<br>2 - G3
Pre-treatment staging Model 1	1 - Stage I<br>2 - Stage II - Stage IV
Pre-treatment staging Model 2	1 - Stage I - Stage II<br>2 - Stage III - Stage IV
Pre-treatment staging Model 3	1 - Stage I - Stage III<br>2 - Stage IV
Post-treatment staging Model 1	1 - Stage I<br>2 - Stage II - Stage IV
Post-treatment staging Model 2	1 - Stage I - Stage II<br>2 - Stage III - Stage IV
Post-treatment staging Model 3	1 - Stage I - Stage III<br>2 - Stage IV
Pre-treatment T staging Model 1	1 - T1<br>2 - T2 - T4
Pre-treatment T staging Model 2	1 - T1 - T2<br>2 - T3 - T4
Pre-treatment T staging Model 3	1 - T1 - T3<br>2 - T4
Pre-treatment N staging Model 1	1 - Nx or N0<br>2 - N1 - N2
Pre-treatment N staging Model 2	1 - Nx or N0 - N1<br>2 - N2

Table S4 Variation rates of blood counts

**Hemoglobin variation**	Hemoglobin level variation in between initial value and maximum drop (in g/dl)<br>Hemoglobin variation rate in time (initial - minimum) (g/dl/day)<br>Hemoglobin variation rate per dose (initial - minimum) (g/dl/Gy)<br>Hemoglobin variation rate per fraction (initial - minimum) (g/dl/day)
**Platelets variation**	Platelets level variation in between initial value and maximum drop (in 10^3/µl; VN: 150-400)<br>Platelets variation rate in time (initial - minimum) (g/dl/day)<br>Platelets variation rate per dose (initial - minimum) (g/dl/Gy)<br>Platelets variation rate per fraction (initial - minimum) (g/dl/day)
**Neutrophils variation**	Neutrophils level variation in between initial value and maximum drop (in 10^3/µl; VN: 2.0-7.7)<br>Neutrophils variation rate in time (initial - minimum) (g/dl/day)<br>Neutrophils variation rate per dose (initial - minimum) (g/dl/Gy)<br>Neutrophils variation rate per fraction (initial - minimum) (g/dl/day)
**Leukocytes variation**	Leukocytes level variation in between initial value and maximum drop (in 10^3/µl; VN: 4-11)<br>Leukocytes variation rate in time (initial - minimum) (g/dl/day)<br>Leukocytes variation rate per dose (initial - minimum) (g/dl/Gy)<br>Leukocytes variation rate per fraction (initial - minimum) (g/dl/day)

Table S5 Calculated mean (and its standard error), standard deviation, variance, extreme values (minimum and maximum) and the 25th, 33rd, 50th, 67th and 75th percentiles of age, weight, BMI for the patients in the study group

		Age (in yrs)	Weight (in kg)	Height (in m)	BMI (in kg/m^2)
Mean		54.19	69.47	4.26	26.33
Std. Error of Mean		1.42	1.73	2.64	0.64
Std. Deviation		10.73	13.06	20.14	4.49
Variance		115.15	170.78	405.61	20.23
Range		48.00	62.00	154.00	20.06
Minimum		28.00	40.00	2.00	15.63
Maximum		76.00	102.00	155	35.69
Percentiles	25	48.50	61.00	1.58	23.23
	33	50.14	63.28	1.60	24.64
	50	53.00	70.00	1.60	26.56
	67	59.00	75.00	1.65	27.85
	75	62.50	76.00	1.65	28.90

Table S6 Calculated mean (and its standard error), standard deviation, variance, extreme values (minimum and maximum) and the 25th, 33rd, 50th, 67th and 75th percentiles of the variables related to the radiotherapy treatment planning and delivery for the patients in the study group

	Planned dose delivery							
	Pelvic region (in Gy)	Dose per raction (in Gy/fraction)	Total fractions	Lombo-aortic lymph nodes volume (in Gy)	Boost dose on lymph nodes (in Gy)	Brachytherapy fractions	Total brachytherapy dose (in Gy)	Radiotherapy halt (in weeks)
Mean	49.54	1.90	26.21	10.91	0.13	2.16	15.90	0.30
Std. Error of Mean	0.21	0.01	0.20	2.69	0.09	0.07	0.59	0.10
Std. Deviation	1.61	0.10	1.51	20.29	0.67	0.53	4.46	0.76
Variance	2.59	0.01	2.28	411.85	0.45	0.28	19.92	0.57
Range	5.40	0.20	4.00	50.40	3.60	4.00	30.00	4.00
Minimum	45.00	1.80	24.00	0.00	0.00	0.00	0.00	0.00
Maximum	50.40	2.00	28.00	50.40	3.60	4.00	30.00	4.00

Table S7 Calculated mean (and its standard error), standard deviation, variance, extreme values (minimum and maximum) and the 25th, 33rd, 50th, 67th and 75th percentiles of the baseline and minimum values recorded during treatment within this study group for hemoglobin, platelets, neutrophils, and leukocytes

		**Hemoglobin**		**Platelets**		**Neutrophils**		**Leukocytes**	
Units		g/dl		$10^3/\mu l$		$10^3/\mu l$		$10^3/\mu l$	
Normal value range		12-18		150-400		2.0-7.7		2.0-7.7	
Level		Initial	Minimal	Initial	Minimal	Initial	Minimal	Initial	Minimal
Mean		12.46	10.63	321.10	183.92	5.74	2.40	8.41	3.43
Std. Error of Mean		0.19	0.19	12.23	8.67	0.29	0.12	0.38	0.20
Std. Deviation		1.49	1.44	93.92	66.56	2.22	0.94	2.95	1.17
Variance		2.21	2.07	8820.82	4430.36	4.93	0.88	8.70	1.38
Range		6.70	7.10	434.00	350.00	9.70	3.60	12.07	4.05
Minimum		8.50	7.50	147.00	45.00	2.00	0.60	3.39	1.62
Maximum		15.20	14.60	581.00	395.00	11.70	4.20	15.46	5.67
Percentiles	25	11.90	246.00	131.00	4.28	1.66	6.44	6.44	2.42
	33	12.20	273.80	147.00	4.55	1.82	6.82	6.82	2.70
	50	12.70	299.00	177.00	5.55	2.27	7.84	7.84	3.28
	67	13.20	347.40	210.00	6.65	2.89	8.94	8.94	4.07
	75	13.50	386.00	233.00	7.43	3.17	10.21	10.21	4.52

Table S8 Calculated mean (and its standard error), standard deviation, variance, extreme values (minimum and maximum) and the 25th, 33rd, 50th, 67th and 75th percentiles of the hemoglobin variation rates for the patients in the study group

		Maximum variation (in g/dl)	Time period (in days) in up to minimal	Time variation rate (g/dl/day)	Per dose variation rate (g/dl/Gy)	Per fraction variation rate (g/dl/fraction)
Mean		1.83	39.07	0.06	0.05	0.10
Std. Error of Mean		0.17	12.59	0.01	0.01	0.01
Std. Deviation		1.34	96.71	0.09	0.05	0.10
Variance		1.80	9352.75	0.01	0.00	0.01
Range		6.50	777.00	0.66	0.29	0.58
Minimum		-1.00	-15.00	-0.40	-0.10	-0.20
Maximum		5.50	762.00	0.26	0.19	0.38
Percentiles	25	0.90	15.00	0.03	0.03	0.05
	33	1.18	22.00	0.05	0.04	0.08
	50	1.80	29.00	0.07	0.05	0.10
	67	2.30	35.00	0.08	0.07	0.13
	75	2.50	36.00	0.09	0.08	0.15

Table S9 Calculated mean (and its standard error), standard deviation, variance, extreme values (minimum and maximum) and the 25th, 33rd, 50th, 67th and 75th percentiles of the platelets variation rates. Normal values interval 150-400×10^3/µl

		Maximum variation (in 10^3/µl)	Time period (in days) in up to minimal	Time variation rate (g/dl/day)	Per dose variation rate (g/dl/Gy)	Per fraction variation rate (g/dl/fraction)
Mean		137.19	25.03	5.61	4.97	9.34
Std. Error of Mean		9.34	1.35	0.53	0.45	0.82
Std. Deviation		71.76	10.24	4.07	3.43	6.23
Variance		5149.88	104.88	16.56	11.79	38.79
Range		432.00	49.00	27.86	22.61	40.70
Minimum		26.00	-7.00	-12.86	0.72	1.30
Maximum		458.00	42.00	15.00	23.33	42.00
Percentiles	25	79.00	15.75	3.47	2.96	5.65
	33	96.20	19.00	4.29	3.51	6.41
	50	133.00	25.50	5.55	4.25	8.14
	67	159.80	32.00	6.37	4.90	9.04
	75	179.00	35.00	6.98	6.47	11.75

Table S10 Calculated mean (and its standard error), standard deviation, variance, extreme values (minimum and maximum) and the 25th, 33rd, 50th, 67th and 75th percentiles of the neutrophils variation rates. Normal values interval 2.2-7.7×10³/μl.

		Maximum variation (in 10^3/μl)	Time period (in days) in up to minimal	Time variation rate (g/dl/day)	Per dose variation rate (g/dl/Gy)	Per fraction variation rate (g/dl/fraction)
Mean		3.33	27.16	0.14	0.11	0.21
Std. Error of Mean		0.27	1.36	0.01	0.01	0.02
Std. Deviation		2.06	10.30	0.09	0.08	0.14
Variance		4.24	106.03	0.01	0.01	0.02
Range		10.10	43.00	0.39	0.39	0.79
Minimum		0.00	0.00	0.01	0.01	0.02
Maximum		10.10	43.00	0.40	0.40	0.80
Percentiles	25	1.74	19.00	0.07	0.06	0.12
	33	2.34	22.00	0.09	0.08	0.14
	50	3.15	29.00	0.12	0.09	0.18
	67	4.09	34.86	0.16	0.12	0.22
	75	4.33	35.00	0.17	0.14	0.26

Table S11 Calculated mean (and its standard error), standard deviation, variance, extreme values (minimum and maximum) and the 25th, 33rd, 50th, 67th and 75th percentiles of the leukocytes variation rates. Normal values interval 4-11×10³/μl.

		Maximum variation (in 10^3/μl)	Time period (in days) in up to minimal	Time variation rate (g/dl/day)	Per dose variation rate (g/dl/Gy)	Per fraction variation rate (g/dl/day)
Mean		4.89	29.22	0.18	0.16	0.30
Std. Error of Mean		0.58	1.30	0.02	0.03	0.05
Std. Deviation		3.33	9.93	0.14	0.15	0.30
Variance		11.09	98.53	0.02	0.02	0.09
Range		11.96	42.00	0.71	0.88	1.77
Minimum		-0.36	7.00	-0.01	-0.01	-0.02
Maximum		11.60	49.00	0.70	0.88	1.75
Percentiles	25	2.59	21.75	0.09	0.07	0.14
	33	3.05	28.00	0.10	0.08	0.15
	50	4.06	29.50	0.17	0.13	0.25
	67	5.90	35.00	0.23	0.20	0.38
	75	7.07	35.00	0.25	0.21	0.41

Table S12 Calculated mean (and its standard error), standard deviation, variance, extreme values (minimum and maximum) and the 25th, 33rd, 50th, 67th and 75th percentiles of the fractions (F) and doses (D) in Gy delivered up to blood test minimum values recorded during treatment

		Hemoglobin		Platelets		Neutrophils		Leukocytes	
		F	D	F	D	F	D	F	D
Mean		17.71	33.32	16.34	30.87	17.53	33.11	18.60	35.38
Std. Error of Mean		1.00	1.84	0.82	1.53	0.88	1.61	0.80	1.46
Std. Deviation		7.64	13.98	6.25	11.68	6.62	12.16	6.06	11.12
Variance		58.32	195.34	39.07	136.35	43.75	147.78	36.77	123.72
Range		26.00	46.80	22.00	39.60	28.00	50.40	22.00	38.40
Minimum		2.00	3.60	5.00	9.00	0.00	0.00	6.00	12.00
Maximum		28.00	50.40	27.00	48.60	28.00	50.40	28.00	50.40
Percentiles	25	10.50	19.35	11.00	19.80	13.00	25.20	14.00	26.00
	33	15.00	29.36	12.00	22.73	14.00	26.28	16.00	30.30
	50	20.00	37.90	16.50	33.00	18.00	36.00	20.00	37.80
	67	23.00	43.20	21.00	39.81	21.86	41.92	22.00	44.00
	75	24.00	45.00	22.00	41.40	23.00	44.00	23.00	45.00

Table S13 Calculated mean (and its standard error), standard deviation, variance, extreme values (minimum and maximum) and the 25th, 33rd, 50th, 67th and 75th percentiles of the pre-treatment and post-treatment MRI tumor volumes

		Pre-treatment		Post-treatment	
MRI Contrast Tumor Volume (cc)		DWI	T2	DWI	T2
Mean		38.13	44.32	0.42	0.39
Std. Error of Mean		13.33	17.40	0.25	0.14
Std. Deviation		32.66	42.61	0.62	0.34
Variance		1066.59	1815.98	0.39	0.12
Range		89.65	112.86	1.59	0.86
Minimum		13.61	16.68	0.00	0.00
Maximum		103.26	129.54	1.59	0.86
Percentiles	25	19.61	21.64	0.00	0.00
	33	23.22	24.11	0.00	0.13
	50	28.33	26.91	0.16	0.44
	67	32.46	38.04	0.52	0.57
	75	51.04	64.33	0.85	0.68

Table S14 Calculated mean (and its standard error), standard deviation, variance, extreme values (minimum and maximum) and the 25th, 33rd, 50th, 67th and 75th percentiles of the rectal V40, V45, V50 (in VMAT and 3D)

		V40 (in %)		V45 (in %)		V50 (in %)	
		VMAT	3D	VMAT	3D	VMAT	3D
Mean		83.84	91.02	70.98	86.18	20.21	54.91
Std. Error of Mean		2.55	1.58	3.65	2.09	2.44	7.23
Std. Deviation		10.80	6.71	15.50	8.88	10.37	30.68
Variance		116.69	45.00	240.23	78.82	107.53	941.27
Range		39.09	19.29	59.18	27.23	35.01	98.78
Minimum		60.88	80.71	40.08	72.77	0.00	0.00
Maximum		99.97	100.00	99.26	100.00	35.01	98.78
Percentiles	25	75.71	83.70	63.43	77.33	10.27	38.35
	33	78.19	87.68	66.94	82.06	15.49	48.28
	50	87.03	91.18	69.91	85.92	21.04	63.27
	67	90.40	96.60	75.22	92.33	27.24	68.42
	75	91.56	96.99	84.44	94.53	28.98	74.22

Table S15 Calculated mean (and its standard error), standard deviation, variance, extreme values (minimum and maximum) and the 25th, 33rd, 50th, 67th and 75th percentiles of the bladder V40, V45, V50 (in VMAT and 3D)

Dosimetric volumes		V40 (%)		V45 (%)		V50 (%)	
RT Technique		VMAT	3D	VMAT	3D	VMAT	3D
Mean		76.46	96.14	60.25	93.53	17.03	68.54
Std. Error of Mean		4.22	1.48	4.22	2.03	2.33	7.35
Std. Deviation		17.39	6.10	17.41	8.35	9.61	30.29
Variance		302.37	37.23	303.00	69.80	92.39	917.47
Range		58.41	22.67	61.54	28.78	34.77	100.00
Minimum		41.59	77.33	29.90	71.22	0.00	0.00
Maximum		100.00	100.00	91.44	100.00	34.77	100.00
Percentiles	25	59.47	94.74	46.02	89.96	10.69	49.90
	33	73.92	96.24	52.91	92.42	11.02	58.70
	50	82.08	99.22	62.00	97.60	15.04	76.79
	67	85.72	99.98	65.13	99.61	15.87	91.99
	75	87.26	100.00	70.15	99.85	22.50	94.20

Table S16 Calculated mean (and its standard error), standard deviation, variance, extreme values (minimum and maximum) and the 25th, 33rd, 50th, 67th and 75th percentiles of the bowel V45 (in %) in VMAT and 3D

RT Technique		VMAT	3D
Mean		217.45	271.33
Std. Error of Mean		26.51	28.72
Std. Deviation		112.46	121.84
Variance		12647.38	14845.08
Range		434.74	475.39
Minimum		30.34	36.06
Maximum		465.08	511.45
Percentiles	25	137.38	196.08
	33	154.61	213.92
	50	222.90	264.28
	67	248.91	348.75
	75	290.10	365.11

Table S17 Calculated mean (and its standard error), standard deviation, variance, extreme values (minimum and maximum) and the 25th, 33rd, 50th, 67th and 75th percentiles of the right and left kidney V18 (in VMAT and 3D)

OAR		Right kidney		Left kidney	
RT Technique		VMAT	3D	VMAT	3D
Mean		25.94	37.50	26.78	28.01
Std. Error of Mean		2.65	3.71	2.27	4.46
Std. Deviation		7.95	11.13	6.82	13.38
Variance		63.18	123.92	46.48	179.10
Range		29.84	40.41	25.39	42.37
Minimum		9.16	14.73	11.20	3.24
Maximum		39.00	55.14	36.59	45.61
Percentiles	25	22.51	31.35	24.89	18.40
	33	24.06	33.56	25.67	27.07
	50	26.79	39.75	28.34	31.31
	67	28.70	40.96	29.30	35.30
	75	29.27	43.14	29.82	35.96

Table S18 Calculated mean (and its standard error), standard deviation, variance, extreme values (minimum and maximum) and the 25th, 33rd, 50th, 67th and 75th percentiles of the mean and maximum dose to be delivered to the right and left kidneys (in VMAT and 3D)

OAR		Right kidney				Left kidney			
Dose		Mean	Mean	Maximum	Maximum	Mean	Mean	Maximum	Maximum
RT Technique		VMAT	3D	VMAT	3D	VMAT	3D	VMAT	3D
Mean		15.24	15.24	48.13	46.56	14.63	12.53	46.63	45.31
Std. Error of Mean		0.60	1.12	1.77	1.80	0.70	1.33	2.09	2.44
Std. Deviation		1.80	3.36	5.32	5.40	2.11	3.98	6.27	7.32
Variance		3.25	11.32	28.27	29.13	4.45	15.83	39.34	53.52
Range		6.50	11.21	16.05	17.20	6.47	11.10	18.51	24.06
Minimum		11.71	8.01	37.80	33.97	10.89	4.67	34.84	28.27
Maximum		18.21	19.22	53.85	51.17	17.36	15.77	53.35	52.33
Percentiles	25	14.05	13.83	44.72	12.88	12.88	9.92	42.16	42.66
	33	14.61	13.96	47.99	13.78	13.78	13.29	44.66	44.38
	50	15.78	15.76	49.64	15.58	15.58	14.29	47.43	48.10
	67	15.89	17.34	50.32	15.98	15.98	14.70	51.26	49.31
	75	15.94	18.11	52.10	16.04	16.04	15.05	52.00	50.36

Table S19 Calculated mean (and its standard error), standard deviation, variance, extreme values (minimum and maximum) and the 25th, 33rd, 50th, 67th and 75th percentiles of the right and left femoral heads V45 and V50 (in VMAT and 3D)

OAR		Right femoral head				Left femoral head			
Dosimetric volume		V45 (in %)		V50 (in %)		V45 (in %)		V50 (in %)	
RT Technique		VMAT	3D	VMAT	3D	VMAT	3D	VMAT	3D
Mean		2.34	8.57	0.00	4.32	1.50	9.40	0.00	4.39
Std. Error of Mean		0.68	2.20	0.00	1.74	0.39	2.21	0.00	1.82
Std. Deviation		2.87	9.34	0.00	7.17	1.67	9.39	0.00	7.74
Variance		8.22	87.32	0.00	51.43	2.79	88.16	0.00	59.88
Range		10.04	33.16	0.01	26.27	5.42	35.31	0.01	29.81
Minimum		0.00	0.00	0.00	0.00	0.00	0.00	0.00	0.00
Maximum		10.04	33.16	0.01	26.27	5.42	35.31	0.01	29.81
Percentiles	25	0.02	2.09	0.00	0.00	0.00	2.15	0.00	0.05
	33	0.06	3.41	0.00	0.00	0.05	4.53	0.00	0.10
	50	1.46	6.14	0.00	1.20	1.05	7.03	0.00	0.95
	67	2.70	9.88	0.00	4.07	2.17	10.39	0.00	3.54
	75	4.01	11.25	0.00	5.46	2.39	12.48	0.00	6.42

Table S20 Calculated mean (and its standard error), standard deviation, variance, extreme values (minimum and maximum) and the 25th, 33rd, 50th, 67th and 75th percentiles of the maximum doses to be delivered to the left and right femoral heads (in VMAT and 3D)

OAR		Left femoral head		Right femoral head	
RT Technique		VMAT	3D	VMAT	3D
Mean		47.73	50.32	47.38	50.64
Std. Error of Mean		0.50	0.56	0.47	0.49
Std. Deviation		2.11	2.38	2.01	2.06
Variance		4.47	5.68	4.03	4.24
Range		6.47	8.83	6.44	7.22
Minimum		44.42	44.22	43.85	45.64
Maximum		50.89	53.05	50.29	52.86
Percentiles	25	45.68	49.60	45.44	50.27
	33	46.52	49.74	46.03	50.49
	50	47.63	50.60	48.18	50.81
	67	49.52	51.95	48.46	51.64
	75	49.71	52.17	48.91	52.41

Table S21 Calculated mean (and its standard error), standard deviation, variance, extreme values (minimum and maximum) and the 25th, 33rd, 50th, 67th and 75th percentiles of the hematogenous marrow V30 and V40 (in VMAT and 3D)

Dosimetric volume		V30 (in %)		V40 (in %)	
RT Technique		VMAT	3D	VMAT	3D
Mean		65.35	80.17	41.59	54.05
Std. Error of Mean		1.73	1.45	1.99	1.86
Std. Deviation		4.59	3.83	5.25	4.92
Variance		21.05	14.69	27.61	24.16
Range		14.16	10.42	14.40	13.20
Minimum		57.96	76.38	34.59	46.26
Maximum		72.12	86.80	48.99	59.46
Percentiles	25	63.06	77.47	37.03	49.58
	33	63.08	77.61	37.48	51.47
	50	65.00	77.85	42.65	54.64
	67	68.07	82.44	44.83	57.66
	75	68.29	82.72	45.83	58.96

Table S22 Calculated mean (and its standard error), standard deviation, variance, extreme values (minimum and maximum) and the 25th, 33rd, 50th, 67th and 75th percentiles of the maximum doses to be delivered to the hematogenous marrow (in VMAT and 3D)

RT Technique		VMAT	3D
Mean		28.13	37.29
Std. Error of Mean		2.36	2.24
Std. Deviation		7.81	7.44
Variance		61.06	55.43
Range		21.12	19.19
Minimum		19.23	29.78
Maximum		40.35	48.97
Percentiles	25	20.36	31.13
	33	21.31	31.62
	50	26.77	33.28
	67	30.26	42.44
	75	35.63	44.94

Table S23 Calculated mean (and its standard error), standard deviation, variance, extreme values (minimum and maximum) and the 25th, 33rd, 50th, 67th and 75th percentiles of the 95% isodose (in VMAT and 3D)

		Cover (in %) VMAT 95%	Cover (in %) 3D 95%
Mean		98.68	98.00
Std. Error of Mean		0.23	0.44
Std. Deviation		0.97	1.89
Variance		0.94	3.56
Range		3.91	8.58
Minimum		95.99	90.86
Maximum		99.90	99.44
Percentiles	25	98.37	98.14
	33	98.42	98.20
	50	98.83	98.48
	67	99.09	98.60
	75	99.38	98.76
